# Chemical Systems for Wetware Artificial Life: Selected Perspectives in Synthetic Cell Research

**DOI:** 10.3390/ijms241814138

**Published:** 2023-09-15

**Authors:** Pasquale Stano

**Affiliations:** Department of Biological and Environmental Sciences and Technologies (DiSTeBA), University of Salento, 73100 Lecce, Italy; pasquale.stano@unisalento.it

**Keywords:** artificial cells, artificial intelligence, artificial life, autopoiesis, chemical AI, cognition, synthetic biology, synthetic cells, semantic information, systems chemistry

## Abstract

The recent and important advances in bottom-up synthetic biology (SB), in particular in the field of the so-called “synthetic cells” (SCs) (or “artificial cells”, or “protocells”), lead us to consider the role of wetware technologies in the “Sciences of Artificial”, where they constitute the third pillar, alongside the more well-known pillars hardware (robotics) and software (Artificial Intelligence, AI). In this article, it will be highlighted how wetware approaches can help to model life and cognition from a unique perspective, complementary to robotics and AI. It is suggested that, through SB, it is possible to explore novel forms of bio-inspired technologies and systems, in particular chemical AI. Furthermore, attention is paid to the concept of semantic information and its quantification, following the strategy recently introduced by Kolchinsky and Wolpert. Semantic information, in turn, is linked to the processes of generation of “meaning”, interpreted here through the lens of autonomy and cognition in artificial systems, emphasizing its role in chemical ones.

## 1. Wetware Artificial Life as the Third Pillar of the Sciences of the Artificial

Artificial Life (AL), the field pioneered by Christopher Langton, who organized the first AL conference in Los Alamos in 1987 [1], is the study of artificial systems that exhibit behaviors characteristic of natural living systems. AL complements the traditional biological sciences concerned with the analysis (“taking apart” approaches) of living organisms by attempting to synthesize lifelike behaviors within artificial media, generally intended for use in computer models and robots. AL contributes to technology by creating artifacts that mimic living systems. At the same time, AL contributes to addressing questions in theoretical biology as it provides a methodology (the “synthetic method”) based on the *understanding-by-building* paradigm, equivalent to saying that lifelike behaviors can be understood by devising generative mechanisms (bottom-up mechanisms). In other words, the interest lies in those artificial systems that are not explicitly endowed with lifelike behavior/patterns/properties. The latter, indeed, must result (emerge) from the local interactional dynamics of the parts that constitute the artificial systems. As evidenced by Langton, AL locates the understanding of life “as we know it” within the larger picture of life “as it could be” [2]. Moreover, all living systems exhibit various forms of cognitive capacities. Therefore, together with the attempts of constructing generative models of living systems, generative models of cognitive systems, as insightfully evidenced by Bedau [3], are also important AL targets. According to the theory of autopoiesis, formulated 50 years ago by the two Chilean scientists Humberto Maturana and Francisco Varela [4,5], the link between life and cognition is strong and deep. In the words of these authors, “living systems are cognitive systems, and living as a process is a process of cognition” ([5], p. 13). Moreover, following Varela, not only autopoietic systems, but also autonomous systems, have mind-like characteristics ([6], p. 270). AL, which targets life and cognition, then becomes one of the most relevant approaches to deepen the current knowledge about two central questions in sciences, i.e., “what is life?” and “what is cognition?”.

As mentioned, the usual methodological approaches developed within AL mainly refer to hardware and software strategies (robotics, computer programs, and especially their combination). On the other hand, AL pioneers also mentioned a third approach: the wetware (chemical) one. AL, because of its bottom-up attitude, “complements the analytic approach of traditional biology with a synthetic approach: rather than studying biological phenomena by taking living organisms apart to see how they work, we attempt to put together systems that behave like living organisms” [2]. Moreover, Langton makes an analogy between the knowledge generated by the processes of synthesis in chemistry (understand chemical phenomena, fabricate new materials and chemicals that are of great practical use for industry and technology) with the knowledge that can be generated via the processes of synthesis in the biological realm (a practice he aptly called “synthetic biology” (SB), anticipating it more than a decade before its birth in the early 2000s). SB, while attempting to generate biological phenomena (self-maintenance, reproduction, adaptation, control, communication, cooperation, evolution, …), contributes to the theoretical understanding of the phenomena under study and to the development of practical applications in biotechnology.

**Figure 1:**
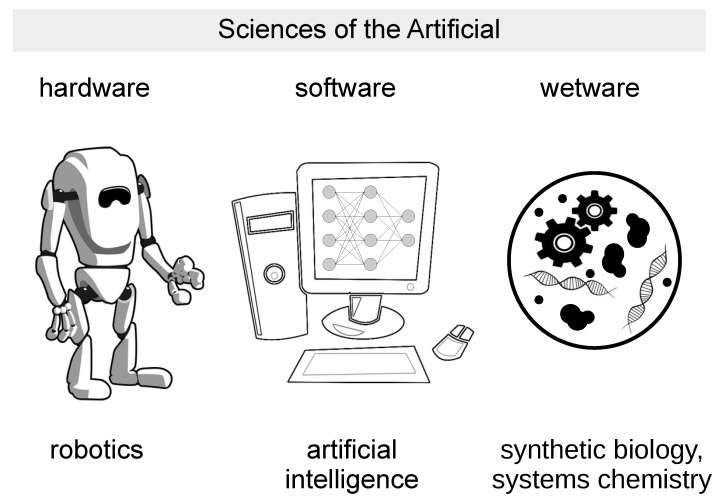
Sciences of the Artificial. The drawing shows three different implementations of artificial models of life, intelligence, cognition: hardware (robotics), software (Artificial Intelligence), wetware (synthetic or artificial cells developed in synthetic biology and systems chemistry).

Some approaches within SB can be considered to be the wetware version of AL, and, in general, one of the three pillars of the “Sciences of the Artificial” [7]; see Figure 1. SB operates in the bio-chemical domain made of molecules, supramolecular systems, colloidal structures and reaction networks, and so offers an unprecedented opportunity to model life and cognition in a way that hardware and software approaches cannot achieve. In particular, wetware models, being physically embodied and thermodynamically constrained, have the possibility of unbound free-energy-driven physical interactions with the world in a manner only partially achieved in robotics, and not achievable by software programs and algorithms. Wetware models are open to all sort of interactions (they can be called “informationally open” [8]) and consequently can be perturbed by (and be capable of adapting to) elements of the environment, in particular by changing the interaction rules of the chemical network in a completely autonomous manner (plasticity). These features, in turn, allow the emergence of meanings and suggest how to overcome the symbol-grounding problem [9,10], a well-known fundamental issue in Artificial Intelligence (AI), and similarly important in AL.

Building, rather than simulating, introduces a radical discrimination about what an artificial system can do. In simulations (e.g., software models), interactions are pre-determined by the designer. Simulations can admittedly generate phenomena that look surprising to the observers. However such phenomena implicitly belong to the set of possible paths predictable a priori once the “rules of the game” are known. Moreover, literally every sort of interaction is made possible in the virtual domain. Constraints can be added, of course, but still they have been set by the designer. However, it is important to remark that the discussions about the predictability of software simulations and the question of whether or not “really new” knowledge can be obtained from them are far from resolved. Any relevant comments on the subject would require, indeed, further and more specialized argumentations which would be far beyond the scope of this article. Building hardware systems is a step forward, as they are embodied systems. The robot body must engage in physical interactions with the surrounding world (e.g., with the ground, with movement constraints, with gravity) and must deal with energy issues to allow movement. However, the macroscopic size of a robot and the materials used for its construction are themselves cutoffs for the kind of physical interactions the robot can experience. When sensors are introduced, e.g., to make the robot responsive to light, or to tactile stimuli, it must be recognized that it is the designer who selects which kind of sensorial inputs the robot should respond to, not the robot itself. On the contrary, wetware AL models, which can be identified as molecular robots built by SB or Systems Chemistry approaches (see Section 2.2 and Section 2.3) because of their structure, are able to experience physical and chemical stimuli because their constitutive elements intrinsically display this property. The latter feature is ultimately due to their microscopic size and to the corresponding small energy exchanges, barriers, trade-offs. As emphasized by Peter M. Hoffmann [11], molecular machines are privileged objects because various forms of energy (thermal, chemical, mechanical, and electrostatic) have the same order of magnitude at the nanoscale and their interconversion is particularly easy at this scale [12].

**Figure 2:**
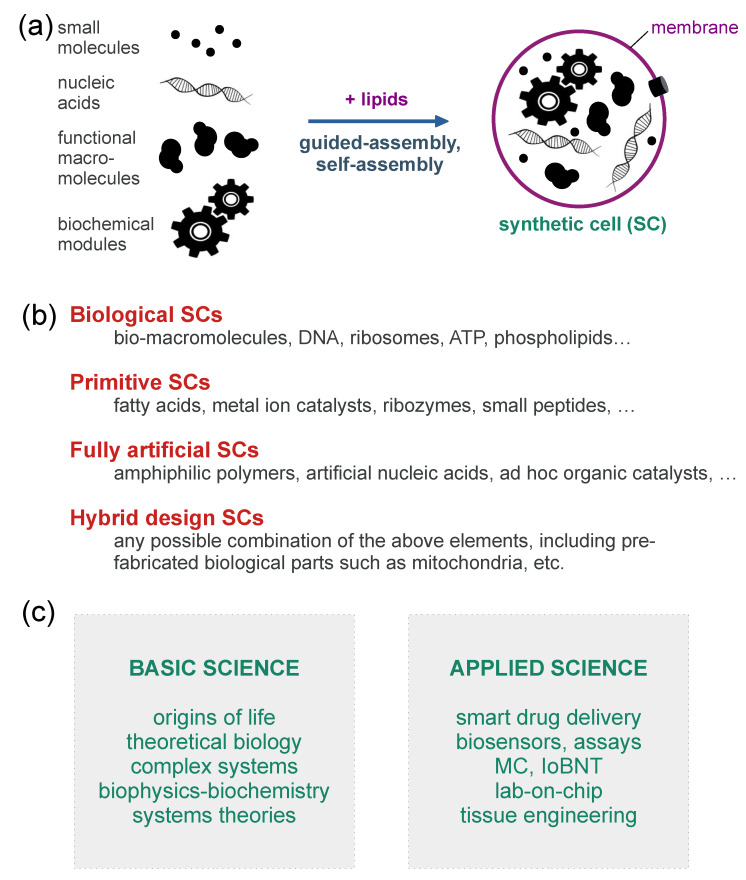
Synthetic cells (SCs) are made by encapsulating (bio)chemicals inside artificial compartments [19,20]. (**a**) Bottom-up construction of SCs from biochemical components. The process is based on a combination of guided-assembly (e.g., the droplet transfer method [21]) and self-assembly (e.g., lipid association in form of bilayers). (**b**) Different types of SCs can be recognized and classified, depending on the materials used for their fabrication. (**c**) Some possible uses of SCs in basic and applied science, the lists not being exhaustive.

The relevance of SB and Systems Chemistry in AL has recently grown. SB, in particular, is the preferred discipline for the construction of artificial living systems. SB encompasses a series of approaches, reviewed in [13], which include (i) the modification of extant organisms by “rewiring” their inner metabolic or genetic regulatory networks, mainly for bio-production and bio-sensing purposes; (ii) the insertion in a living cell of a whole genome, having minimal size and generated in artificial manner, in order to explore the minimal complexity of cellular life; (iii) various cell-free methods, including in vitro protein synthesis, biochemical reconstitutions, and micro-compartmentation technologies. The latter techniques, and others, allow the construction of synthetic or artificial cells (SCs/ACs) from scratch (Figure 2a), aiming at (a) developing molecular, supramolecular, or cell-like biotechnological tools; (b) constructing simplified models of living cells; (c) exploring the emergence and the origin of life in the laboratory (some types of SCs/ACs are recognized as “protocells”, i.e., primitive cell models). The “explorative” vein of SB, which is particularly evident in point (iii-b) and (iii-c), overlaps with many goals, experimental methods and theoretical frameworks of Systems Chemistry [14,15,16,17,18]. Systems Chemistry is a new branch of chemistry that, stemming from origin-of-life and prebiotic chemistry, aims at understanding a large set of sophisticated chemical phenomena such as self-assembly, compartmentalization, self-organization, non-linear dynamics, oscillations, autocatalysis, adaptivity, evolution, and so on. It focuses on many sorts of chemical systems, including cell-like ones. Systems Chemistry adopts the understanding-by-building approach.

The present article is dedicated to the illustration of some recent trends in wetware AL, SB and Systems Chemistry, focusing on SC research. SCs are simplified models of cells—not yet alive to date—that can be built in the laboratory according to modern biotechnological methods mostly based on cell-free systems, liposome technology, microfluidics, and numerical simulations (Section 2). A fascinating problem for the conceptualization of “what SCs actually are” is the here-proposed distinction (and the related question about whether or not it is correctly posed; see also [22,23]) between machine-like and organism-like SCs. From the discussion and the perspective we propose, it will become clear that the distinction can be mainly related to the capacity to allow bottom-up emergence of meaning in the chemical systems we call SCs. We will shortly present the first moves of a recently promoted theoretically oriented direction, namely the utilization of SC scenarios as tools for defining and quantifying the “semantic information” SCs find in their environment (Section 3). The focus on semantic information (also called functional, meaningful, relevant, or pragmatic information—see cited references in [24]) and its origin in biological systems is an opportunity to (i) re-discover the possible approaches put forward by theoretical biologists, and (ii) translate these approaches into a set of questions: can AL wetware systems, in particular SCs, self-generate meaning? Which systems? To what extent? In which conditions? With what constraints? A brief final comment summarizes the discussion and highlights perspectives for future studies (Section 4).

## 2. Synthetic Cells as Tools for Exploring Artificial Life and Artificial Intelligence

A brief introduction to bottom-up SCs and more details about experiments that will be relevant to the present discussion are provided in Appendix A, which is dedicated to readers who are less familiar with the subject. To summarize, bottom-up SCs can be defined as human-made molecular systems partially resembling biological cells both in structure and function. They can be constructed by employing various types of chemicals, such as proteins, nucleic acids, lipids, and metabolites, but also allegedly primitive compounds (e.g., fatty acids, short peptides, ribozymes) or non-biological ones (e.g., polymers, ad hoc designed catalysts, artificial lipid-like compounds, unnatural nucleic acids); see Figure 2b. The resulting structures are micro-compartmentalized chemical systems (e.g., ca. 0.1–10 μm), to date still far from being alive, but that display interesting lifelike behavior [25,26,27,28]. SCs can be seen as very simple systems if compared to biological cells, but at the same time quite complicated systems if compared to ordinary chemical reactions occurring in the test tube. Confinement, crowding, the presence of a soft interface, the semi-permeability of the SC boundary, and the high surface-to-volume ratio make SC behavior particularly interesting from a chemical viewpoint.

It is important to emphasize that the prefix “bottom-up” identifies the kind of SCs we are referring to (Figure 2a). In fact, the name “SCs” also applies to other structures obtained by completely different methods. For example, the modifications of extant living cells, such as the addition, elimination, integration, combination of genetic elements, or even the whole-genome transplantation [29,30], generate other types of SCs. The present article is dedicated only to bottom-up SCs. We intend to highlight their role, as wetware AL systems, in modeling life and cognition. The reason is that theoretical and experimental bottom-up approaches match AL philosophy and perspectives rather well. In this way, we hope the discussion will lay the groundwork for innovative investigations, directing the reader’s attention to specific and unexplored aspects of what SCs are and how they behave.

For the reasons indicated in Section 1, bottom-up SCs are tools that would have been of certain interest in the early days of cybernetics. Wetware systems would have been candidates for inquiring life and cognition via the construction of artificial systems. To quote Arturo Rosenblueth, Norbert Wiener and Julian Bigelow, “In future years, as the knowledge of colloids and protein increases, future engineers may attempt the design of robots not only with a behavior, but also with a structure similar to that of a mammal” ([31], p. 21); or, to quote Donald M. MacKay, “[…] one would have to go in for mechanisms in protoplasm instead of mechanisms in copper” ([32], p. 221).

Bottom-up SCs are embodied agents (as robots are), and can also be situated agents when placed in a dynamical environment with which they can interact. Bottom-up SCs, then, might become an interesting and possibly far-reaching platform in AL and AI. They should be built in order to model life and cognition [33,34] and the emergent properties at the bare minimal complexity level, thus being directly connected to the origin of these features in primitive/minimal life. The form of AI that SCs can target is not the same as the equivalent concepts, usually referred to as brains, in higher organisms. We refer, instead, to basic features associated with intelligence, in particular, what is the meaning of “knowing”, e.g., the environment, the external world, i.e., coping with the set of disturbances that a system perceives. It should be noted that “disturbances” (reasonably corresponding to “perturbations”, also used in this article, and hereby considered synonymous) was the term used by W. Ross Ashby for explaining concepts of stability and ultrastability in [35,36]. For example, a possibility is to scale down to the level of uni- or pauci-cellular systems, concepts or questions already elaborated upon in AI, such as the Turing test. The test, also known as the imitation game, was originally formulated in the domain of human language [37], but it has been recently reformulated for the wetware domain of chemical SCs [38] (and, as such, already referred in experimental investigations [25]; see the comments in [39]).

### 2.1. Recognizing the Twofold Value of Wetware Approaches

It seems to us that the employment of SCs in AL and AI represents a broad opportunity for exploring, and perhaps re-discovering, many concepts related to basic and fundamental questions that have been mainly discussed in robotics and AI in past decades and in contemporary times. Yet, wetware systems have the potential of facing these questions in an innovative and fruitful manner. The wetware approach can provide hints and disclosures whose usefulness is somehow more basic [40]. In recent years, we have been involved in the proposal of SB approaches to forward AI, in particular by proposing a specific form of embodied AI called “autopoietic (or organizationally grounded) embodied AI” [33,41,42] which resonates with the current discussion. In short, the research program aims at overcoming some limitations of “Organismically inspired Robotics” and “Enactive AI” [43,44] by bringing the autopoietic organization onto the experimental scene through a wetware autopoietic approach to embodied AI. This translates into wetware-based approaches that rely on chemical networks that are defined and that propagate in the functional space and in the structural space, self-generating autopoietic and embodied agents [41].

The emphasis we have placed, till this point of the discussion, on the role of bottom-up SCs in basic science should not be mistaken for a lack of interest in applications (see Figure 2c). The field of nano-medicine has been already identified as one of the most appropriate for developing new SC-based biotechnology due to the potential use of SCs as “smart” drug delivery agents [45,46,47,48], as biosensors, as tools for bioassays [49]. Bottom-up SCs (and SCs in general) can also play a decisive role in the frontier scenario called the “Internet of Bio-Nano Things” (IoBNT) [13]. The latter has been put forward by communication engineers interested in Molecular Communication (MC), and focuses on nano- and microscale artificial elements (parts, devices, systems) communicating with each other. The resulting bio-nano network should be designed and constructed to achieve specific functions when these artificial elements, interfaced with living cells, behave in a coordinated manner, in particular inside the human body [50,51]. For example, SCs should be capable of exchanging information between each other and with biological cells in a predictable manner. The underlying enabling technology, called MC, has been pioneered by Tadashi Nakano in the 2000s [52,53] and can play a synergic role, together with SB, for the advent of these futuristic perspectives.

It is therefore evident that for some scopes, SCs must behave as molecular robots (*machine-like* SCs) because they need to be fully programmable, and their behavior should be foreseen with accuracy. They must obey a sort of program (or algorithm) that is embodied in the chemical network they are made of. The designer must be able to predict their behavior. On the other hand, and for other scopes, SCs should display lifelike properties and rather tend toward forms of autonomy (and, ultimately, become alive). These kinds of *organism-like* SCs are instead particularly relevant for addressing basic questions about the emergence of life, the living organization, and become privileged experimental models of life and cognition. Needless to say, all dichotomies rarely fit to reality, and it is often possible to identify both machine-like and organism-like traits in actual SCs—the ones currently built in laboratories. Let us briefly discuss, however, these two connotations separately, because it is simpler to highlight their characteristic features and provide additional considerations.

### 2.2. Machine-like SCs

SCs can be constructed to perform some predetermined (and useful) function, e.g., they can operate as “smart” drug-producing or drug-delivery agents. In this case, the scope of the bioengineer is to produce SCs with limited between-SC variability which are easily programmable and display fully controlled behavior. In a sense, they can be conceived as molecular robots, having internal chemical machinery with a goal that is fixed in advance, e.g., the production of a drug. The functioning of the chemical machinery is of course important: everything is functional to a globally linear input–output logic. Given a set of external signals, for instance, a drug must be produced in the right spatial-temporal location. Global behavior can be defined “responsive” or “reactive”, i.e., stimulus-response behavior. Using the language of autopoiesis [5], such a system is *allopoietic*: it produces something that is not itself. Moreover, the designer of machine-like SCs decides in advance to which signals SCs must respond. These signals have a “meaning” for the designer, e.g., cancer cell exudates as a sign of the presence of cancer cells. The SC inner machinery is intended as a fixed “map” [54] of the environment it will be situated in, but the map has not emerged from the SC function but from the ingenuity of the designer. Machine-like SCs are born without needing to learn. Machine-like SCs can share some aspects of living systems, but in general, they are not built to be alive.

According to this vision, the concept of “chemical AI” in machine-like SCs can be considered an extension of the computationalistic AI in the chemical domain. For example, chemical concentrations play the role of data, and chemical reactions play the role of operations. Thanks to the peculiarity of the computational medium (the chemical network), computation is carried out in parallel, a feature which per se is highly relevant for chemical AI. Discussions about how to implement chemical AI are already present in the scientific literature, which generally does not refer to SCs, but to non-compartmentalized chemical systems. Phrases such as “unconventional computing” or “natural computing” are also used. In this respect, it is useful to recall that any distinguishable physicochemical state of matter and energy can be used to encode information, and every natural transformation of this state is a kind of computation. In particular, it is possible to exploit the physicochemical laws to make computations. Every physicochemical law describes a causal event, and any causal event can be conceived as a computation. The causes are the inputs, the effects are the outputs, and the law governing the transformation is the algorithm of the computation [55].

In the context of chemical AI for SCs, one can conceive the whole chemical reaction network embedded in the SC compartment as a computational network. Since the computing elements are mainly proteins (sensors, enzymes, transcription factors) [56], very interesting behavior can emerge, because most proteins are conformationally dynamical, and also often subjected to allosteric regulation—a feature which makes proteins approximately similar to transistors [57]). In addition, the use of mutants can play a key role in the design and engineering of chemical networks with tunable properties. In cellular physiology, signaling networks and gene regulatory networks have neural-network-like features [58], and SB has often borrowed this concept to design, simulate [59], and sometimes build chemical artificial neural networks [60], either working on the DNA displacement principle [61] or as signaling pathways for SCs [62,63,64,65,66,67,68]. Much more work is required in this direction to extend these early attempts to other sorts of computational systems (e.g., Bayesian—see below).

Owing to the overall allopoietic dynamics that characterize the linear logic of these types of processes (even if it might include cyclic “subroutines”), this chemical computation is not a self-computation (it does not lead the system itself, i.e., it does not generate the system itself as a product of its own computations). Therefore, the system cannot autonomously assign meaning to signals perceived from the external world, such as what is good or not for its own existence [69].

### 2.3. Organism-like SCs

True organism-like SCs are not easy to build, and indeed have not yet been built. But in this category, we can place those SCs whose design is at least planned to generate lifelike behavior for its own sake rather than to achieve other functions. This is the realm of research devoted to constructing minimal living systems, based or not on an origin-of-life scenario. We would call these SCs *autopoietic* (self-producing), and therefore they would be also cognitive and alive. Implicitly, they will be endowed with the capacity of self-organization. Because the construction of an autopoietic SC is difficult, a possible intermediate goal, still in the category of organism-like SCs, is the implementation of *autonomous* behavior, a key feature for helping to advance wetware AL. Varela has defined autonomous systems in the same way as autopoietic ones, but does not include the important condition of only focusing on “production” processes ([6], p. 55). Autonomous systems are characterized by networks of processes that recursively depend on each other in the generation and realization of the processes themselves, and that constitute the system as united and recognizable in the domain in which the processes exist. The definition requires several additional specifications that cannot be given here (interested readers should refer to [6]). For the sake of the present discussion, we can focus on the idea that even if currently affordable organism-like SCs are not autopoietic, efforts should be made to make them at least autonomous. The way to do so, however, is not specified, and it is indeed open to discussions, proposals, verification, and criticisms. The processes that first come to mind for the construction of autonomous organism-like SCs are chemical networks, where the processes are linked to each other by entailment relations (recently, we have proposed a route based on recurrent chemical neural networks [67,70]).

Importantly, Varela proposed that autonomous systems are also mind-like systems ([6], p. 270) because they can distinguish self and non-self, and because they are cognitive in the sense specified above (Section 1, i.e., when the environment “in-forms” the system [22], which, owing to its plasticity, becomes “in-formed” by compensating the perturbations exerted by environmental factors). As a result of these operations, known as “structural coupling” (and following other interpretations, with somehow different meanings, “enaction” [71], identification of “affordances” [69,72], and generation of the *Umwelt* [73]), the system knows, or learns, the environment; in this sense, specific environmental factors become meaningful. (For these parallelisms—here mentioned in an oversimplified manner—I am indebted to J. C. Letelier (University of Chile) for his talk entitled “Diagrams to understand Structural Coupling” presented at the workshop “SB-AI 8. What can Synthetic Biology offer to Artificial Intelligence? Strategies and Perspectives for Embodied Chemical Approaches to AI” (ALIFE 2023 Conference, 24–28th July, 2023; Sapporo, Japan)). Remarkably, the meaning originated from such a dynamic process has an intrinsic *relational* nature.

The correspondence, advocated for by Varela, between autonomous and mind-like systems opens a broad perspective for SB-based modeling in cognitive sciences and in AL. A productive cross-fertilization and a source of inspiration can then stem from models of brain functioning (with proper modifications, of course) such as the ones based on Bayesian inference, Helmholtz machines [74,75,76,77], the free energy principle [78,79], and possibly “autonomatic” mechanisms [80]. Because of the very simple structure bottom-up SCs have when compared to biological cells, the applications of these models, e.g., in defining conditional probability matrixes in Bayesian approaches, will possibly be within experimental/modeling reach.

From the viewpoint of semantic information, which will be discussed later in more detail, it is expected that organism-like SCs self-generate meaning according to their self-maintenance dynamics, thanks to the possibility of adapting to the environmental stimuli (seen as perturbations), and change their organization accordingly. The goal is to understand self-organization mechanisms that lead to the emergence of the meaning directly from the analysis of the system dynamics. Organism-like SCs should display plasticity in their dynamic organization. The question, again, is whether at least partial forms of these processes are realistically possible with current SC technology. Future research can be inspired by previously published considerations, such as [69,81,82,83,84,85], adapting them to SC dynamics.

The concept of “chemical AI” in organism-like SCs has been approached by us in a recent proposal, called “autopoietic (or organizationally grounded) embodied AI”, already mentioned in Section 2.1. In addition to its conceptualization and to the definition of programmatic lines (which wait, however, for concrete realizations), the organizationally based approach has led to a taxonomy of artificial systems [34], and to the perspective of “living technologies”, which is quite resonant with what was already depicted by pioneers in this field [86,87].

### 2.4. Between Machine-like and Organism-like SCs

It is useful, at this point, to identify additional arguments that alleviate the tension of the machine-like vs. organism-like dichotomy.

Are machine-like SCs really so stiff, informationally closed, and therefore fully predictable? Certainly, these properties fall within the scope of the bioengineer (to perform specific functions and be programmable and realizable in a highly controlled way). To verify that the SC behavior complies with these requests, it is first necessary to remove the stochastic sources of unpredictability. Some bottom-up SB technologies can help to reduce extrinsic stochastic factors (between-SC variability [88]), for example, by microfluidic-assisted guided assembly. However, intrinsic factors still remain and will produce variability given a defined network structure. But the chemical network itself, even if designed (and expected) to follow uniquely (pre)defined dynamics (i.e., we can say, being informationally closed), cannot avoid unwanted interactions. Molecules are not mechanical pieces (Section 1) and reactions are not wires. Consequently, molecules have access to a potentially unlimited space of interactions, whose “dimensions” are not restricted by any law (rather, they can evolve as a result of previous interactions), but perhaps can be restricted by tight control of the SC environment. As a consequence, machine-like SCs can display fully predictable behavior only if the environment is also under experimental control. If not, machine-like predictability can fail, showing the limit of the cell/electronic circuit analogy so dear to SB. The design of machine-like SCs has strong analogies to the philosophy of first-order cybernetics: it is the practice of controlling artificial systems in order to reach pre-determined goals. However, it is technically more difficult due to the unlimited allowed interactions that are possible in the molecular world (and that pave the way to perturbations and resulting adaptive response, leading to organism-like behavior). For machine-like SCs, using concepts related to information theory appears useful and adequate [89]. Moreover, the tools provided by the MC theoretical framework are functional to reach high standards in controlling SCs.

The mirroring considerations, now referring to organism-like SCs, are as follows. It is perhaps too ungenerous to reduce all currently developed SCs to the status of machine-like systems and exclude the possibility of glimpsing, already, at least partial aspects of organism-likeness in current SCs. Despite the limited organism-like functions developed so far, SCs are still chemical systems, and as such, they are in principle (and actually) freely open to being perturbed by any entity in the physicochemical domain. Overt manifestations of such openness are found every time the reaction network under inquiry is subjected to capricious variations of its “performance” due to the presence of other chemical entities that do perturb the network of interest. Examples are additional molecules, modules, and circuitries that the designer has introduced for expanding SC functions. One of the research goals of SCs, when conceived as organism-like systems, would be to explore whether the networks of interest already have the potential to display degrees of plasticity, intended as changes to their dynamical organization that make them compatible with new perturbations, are essentially capable to cope with them, and yet maintain overall dynamical stability. Looking at extant SCs from this new perspective will possibly provide an early recognition of organism-like traits in current SCs. This new perspective, however, needs more specifications and constraints in order to avoid every minor disturbance being recognized as a self-organization-triggering event. In other words, it is not enough to focus on traditional goals such as the self-production of all SC components, but cognitive aspects should also be considered. They are rarely discussed in contemporary publications on SC research [90]: summarized in a motto, working on *minimal cognition*. Finally, it must be added that the idea of open-ended self-organization, adaptation, and evolution lies on the top of another implicit idea, i.e., that the environment is also unconstrained and non-designed, and this is clearly not true in typical scientific experiments. But, strictly speaking, only in this case will the environmental perturbations (and the consequent series of learning events) freely unfold, unpredictably, giving rise to unique historical paths [91,92,93].

## 3. Estimating Semantic Information in Situated Synthetic Cells: A Case Study

We have mentioned that the concept of information and its meaning are related to the symbol-grounding problem mentioned in Section 1. This is a relevant and expandable research area that SC investigators are recently facing [94]. It can be applied to analyzing the behavior of machine-like and organism-like SCs.

The usual way to scientifically conceptualize information refers to the definition given by Claude Shannon in the 1940s, firmly related to the problem of communication of messages. Information has a well-known numerical value, probabilistic in its essence, which is associated with the reduction in uncertainty about messages emitted by a source after their observation at the receiver. Information, intended in this sense, can be measured in a rigorous objective manner, but it was not conceived to tell anything about the meaning of messages. Shannon information theory is, essentially, a signaling theory. The word information, on the other hand, literally means “to shape, give form to” (from Latin *in* + *formare*), i.e., generating a change in the receiver to account for some aspects of reality, unknown before. Theories about these aspects of information, which can be defined as “semantic”, were already present in the early days of cybernetics [94,95,96,97,98], especially in the so-called British school [99]. MacKay pointed out that semantic information is related to what information “does” when received by an agent. More precisely, it has a “selective function on the range of the recipient’s states of conditional readiness for goal-directed activity” [81]. Semantic information, then, would be related to the changes that a message causes in the internal organization of the receiver, with the consequence of modifying its (future) behavior. Broadly speaking, semantic aspects of information can be considered as an instance of self-organization in the agent/environment super system.

Early discussions on semantic information focused on human communications and languages [100], and therefore are not properly adaptable to physico-chemical systems like SCs. On the other hand, it seems to us that SCs and similar experimental platforms represent an opportunity for inquiring about minimal forms of semantic information in an unprecedented manner. Contrary to human communication, which largely occurs via languaging, communication in the molecular context of biological cells and SCs typically occurs in the chemical domain (presence/absence of a certain chemical, concentration gradients, diffusion, directed transport, appearance and disappearance of chemical species upon chemical reactions) and occurs both within and between cells. For a recipient cell, the environment is the source of signals (inputs), while the set of processes that molecular signals “activate” can be considered as the physical communication channel [101]. As a result of signal processing (and stochastic noise too), variations of chemical concentrations in the system are generated, and can be considered as outputs. The question becomes whether it is possible to understand where and how semantic information is generated. It must be said that a literature search provides many discussions about semantic information at a high level, especially in the epistemology area, while attempts to provide an operative definition of semantic information, and therefore a route for its quantification, are rare. In Section 3.1 and Section 3.2, a recent proposal will be presented and illustrated with one example tailored to SCs and a realistic environment. In Section 3.3, the example will be generalized in order to grasp how the so-calculated semantic information can contribute to the above-mentioned discussions on the emergence of meaning and to approach the symbol-grounding problem in SC contexts.

### 3.1. The Kolchinsky–Wolpert Approach

Kolchinsky and Wolpert (KW) have recently proposed a quantitative measure of semantic information defined as “the information a physical system has about its environment that is causally necessary for the system to maintain its own existence over time” [24]. It means that semantic information is the *causally necessary part* of the total syntactic information (exchanged with the environment) that the system needs to acquire—considering the system as the information receiver—in order to use it to preserve its privileged out-of-equilibrium state. This definition has been tailored for any out-of-equilibrium system, including non-living ones, and therefore can be applied (i) to living systems of any sort, including living SCs when they will be available, and (ii) to non-living SCs operating out of equilibrium. In other words, the basic idea is that the inevitable dynamics, which lead any out-of-equilibrium system toward thermodynamic equilibrium, must be counteracted by some processes that correspond, from the viewpoint of the observer, to an acquisition of information. Said in another manner, being out of equilibrium means residing in a low entropy state: to keep this state, the system needs to feed itself with information (traditionally identified as negentropy [102]). Note that the phrases “acquisition of information” or “feeding with information” used here imply that information is a *thing*. This concept largely fits with the computational paradigm of cognitive sciences (cognitivism). The perspective we promote, instead, is based on self-organization and self-regulation and considers information as a *process*, i.e., the process of being informed (i.e., in-formed). To quantify semantic information, however, KW utilized the computable metrics of (syntactic) information theory and therefore it is convenient to continue to use that language. As anticipated in Section 4, future work needs to be devoted to identifying other semantic information approaches that could be based on different grounds and described by an alternative language. The KW approach has the merit of being quantitative, rooted in modeling physical systems such as situated dynamical systems, and potentially applicable to wetware AL systems such as SCs.

The model is based on the calculation of information-theoretical quantities, such as the mutual information *I* (for the “stored” semantic information) or the transfer entropy TE (for the “observed” semantic information), both referred to as the interactions between the states of the environment and those of the system, generally considered for simplicity as first-order Markovian stochastic dynamics. For example, in the case of stored semantic information, the mutual information *I* is calculated with respect to a communication channel made of (i) the distribution of the environmental signal intended as the input, (ii) the inner SC mechanism (acting as a transformation operator), and (iii) the distribution of one (or more) SC variable(s) intended as the output. The latter serves to compute the value of the viability function *V* at any time and, in turn, the degree of existence (in a far-from-equilibrium state) of the system. KW devised the following strategy to measure semantic information. Given a certain spatial-temporal distribution of the environmental variables, which assures the best conditions for the system viability, the distribution is subjected to various coarse-graining functions, resulting in a degradation of (and reduction in) the information exchanged between the environment and systems. These variations, defined as *interventions*, are characterized by different syntactic information and might lead, or not, to a reduction in the system viability. Various interventions are evaluated. For each one, the corresponding viability of the system is calculated. The goal is to find a particular intervention (that KW called “optimal”) that does not reduce the system viability while keeping the syntactic information at a maximal value. Semantic information (in bits) is numerically equal to the syntactic information exchanged between the system and the environment under the optimal intervention.

### 3.2. Stored and Observed Semantic Information Calculated for a Situated SC

**Figure 3:**
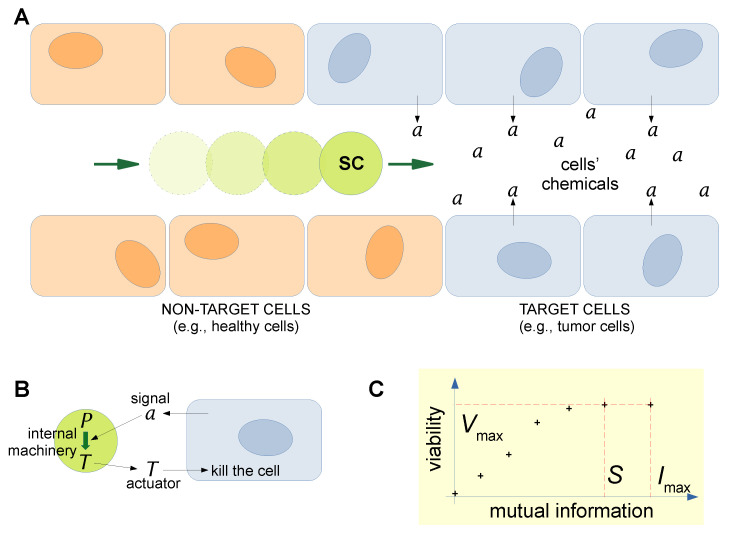
A realistic scenario of SCs as “smart” drug delivery systems and their possible use as a model for quantifying semantic information. (**A**) An SC—endowed with sensors, controllers, and actuators—travels in a blood vessel to reach a region with a disease. The target cells, owing to their dysfunctional metabolism, produce a disease-specific chemical *a*. (**B**) A communication channel then takes place between the dysfunctional cells and the SCs, whereby the signal a triggers the production, inside the SCs, of a drug/toxin *T*, which is released in order to kill (or heal) the target cells. The SC is the system we are interested in, also indicated as **S** in the main text. The cancerous cells, on the other hand, represent the environment wherein SCs operate. Such an environment, irrespective of its actual structure, will be indicated as E in the main text—it is just what is around the system **S**; in other words, the environment is the complement to **S**, or the “not-**S**”. The union of a system **S** and its environment **E** will be called super system **S-E**. (**C**) This “smart” drug delivery scenario has been analyzed in terms of semantic information, as defined by Kolchinsky and Wolpert [24]. The drawing aims at pictographically explaining how the semantic information *S* is a fraction of the maximal mutual information *I* exchanged between a given system **S** and an environment **E**. By definition, *I_max_* corresponds to a situation where the value of the viability function is also maximal *V_max_*. The cross-points represent couples of values computed for each applied intervention. In particular, the semantic information S is the part of mutual information I exchanged between the environment (the target cells) and the agent (the SC), which is strictly necessary not to decrease the agent “viability” *V_max_*. In a “smart” drug delivery context, the viability can be defined as the capacity to complete the task of sensing a and producing *T* (in other contexts, viability can refer to other relevant behaviors, including SC self-maintenance). Technical details in [103,104]. Reprinted with permission from Ref. [13]. Copyright 2023, Authors of the article.

In order to apply the KW approach, we have recently devised a scenario inspired by current research on the utilization of SCs as “smart” drug delivery agents, as noted in Section 1 and Section 2.2: an SC is situated in a capillary, traveling toward cancer cells—see Figure 3A. Such a hypothetical SC (the system of interest, abbreviated as **S**) can be designed in a realistic way, taking into account the current possibilities offered by SC technology. In particular, the SC produces a toxin *T* when it receives a chemical signal *a* from cancer cells, the latter representing its environment (abbreviated as **E**). The processes taking place in the overall super system made by the SC and its environment (abbreviated as **S-E**), shown in Figure 3B. Thanks to dedicated internal machineries (e.g., receptor, response regulator, DNA transcription and translation (TX-TL), etc.), SC can produce and release a toxin protein *T* as a response to the presence of the signal *a* released by the cancer cell. The toxin kills the cancer cells. Note that this SC is a machine-like allopoietic system: the success in toxin production can be taken as a measure of SC viability in that specific environment, and therefore, in this case, what is evaluated is the capability of performing the pre-defined goal for which the SC has been built. However, the approach can be made more similar to the original KW one if extended to the more interesting case of organism-like SCs, instead evaluating the SC’s very existence. The scenario depicted in Figure 3A,B has been the starting point for the calculation of stored and observed semantic information, as detailed in [103,104,105]. The model has been further simplified by removing the spatial dimension of Figure 3A, i.e., by placing a static SC in an environment that is just characterized by the concentration of *a*. The SC dynamics have been modeled by some simple algorithmic rules that mirror the finer patterns generated by sets of differential equations usually employed to describe SC’s behavior [106,107]. Details about the procedure can be found in [24,103,104,105]. The required information-theoretical quantities for the original and the intervened distributions have been calculated, each with an associated value of SC viability. Graphs like the one shown in Figure 3C have been obtained, allowing us to estimate semantic information (in bits). For instance, while the mutual information between the system **S** (the SC) and the environment **E** is 2.32 bits, the semantic part is only 1.92 bits. For the sake of current discussion, the important point is that given a specific **S-E** super system, characterized by a distribution of variables in **E** and a set of rules for determining the dynamics in **S**, it is possible to quantify the part of total syntactic information that “counts” (or that is meaningful) for the systems **S**. The next question is about how to understand the calculated values of semantic information with respect to the above-mentioned issues of meaning and its origin in natural and artificial systems.

### 3.3. Towards the Understanding of Semantic Information and Meaning for SCs

**Figure 4:**
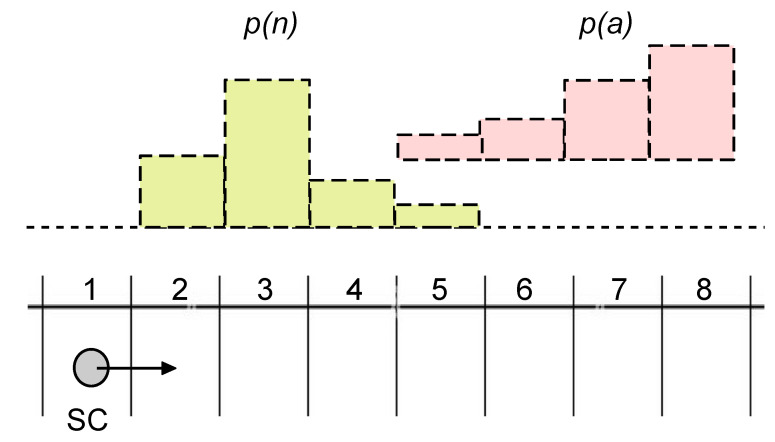
A synthetic cell (SC) situated in an environment. The environment is made by eight discrete locations, numbered from 1 to 8, where the SC moves (from 1 to 8), residing in each location for a certain amount of time. The nutrient molecule *n* and the signal molecule *a* can be found in locations 2-3-4-5 and 5-6-7-8, respectively, with probability distributions p(n) and p(a), sketched above as a bell-shaped and ramp-shaped distribution. Depending on their organization $\Omega_{i}$, different SC${}_{i}$ will display different internal dynamics (manuscript in preparation).

In Figure 3A, we have described a scenario where an SC (the system **S**) is situated in an environment **E**, which is potentially rich in information distributed in time and space. However, our previous study on the determination of semantic information [103] has been limited to a very simple case, just to show that the KW approach can be successfully applied to SCs. The original example, indeed, was about food-caching birds (the system) in the forest (the environment). In order to develop the argument we are interested in, it is necessary to take a step further and consider more complex situations because it will be easier to imagine how *diverse* SCs would behave. Just to make an example, consider a scenario, still inspired by Figure 3A, where the **E** states are specified by the combination of two variables (e.g., a nutrient molecule *n* and a signal molecule *a*). Moreover, these variables can have a spatio-temporal distribution, as shown in Figure 4. The situation refers to an SC traveling in the blood flow, meeting *n* and *a* molecules. p(n) and p(a) are their distributions. In this example, the two distributions overlap, generating further complexity because in some locations the environment could present two stimuli simultaneously. Clearly, more complicated **E** can be similarly conceived. For the sake of the present discussion, the exact scenario we are referring to is not important. The important point is that, in general, the environment **E** is more complex than the one previously analyzed [103]. This complexification has relevant consequences for developing the main argument of this Section as shown below.

SC’s internal circuitry in more complex environments is also required to be more complex. This translates into a more complex set of rules that determines the SC’s dynamics (the SC behavior). For example, SCs whose internal dynamics are optimal for facing two distant p(n) and p(a) distributions cannot be optimal when *n* and *a* are present in the same location at the same time. Moreover, the output variables that co-determine the SC state and the value of the viability function necessarily become more numerous and are probably related to each other by non-linear dependencies. This sharply contrasts with the simple case reported above, where SC viability was assessed just by its capability of producing the toxin *T*. For example, in addition to the amount of toxin produced, it would be necessary that the SC produces internal high-energy biochemical compounds to fuel synthetic modules. In general, as SCs become less machine-like and more organism-like, defining their optimal states will become more complicated. Ultimately, the interest will focus only on the high-level property of “being or remaining alive”. In any case, we can imagine that once defined, the viability function can be computed and therefore it will be possible to decide whether or not the SC state falls in a specific viability region $R_{V}$, in the state space, compatible with a behavior of interest.

Let us call “organization” Ω the set of rules or mechanistic specifications that correspond to the functioning of the SC reaction network. Ω implicitly or explicitly includes the topological relations between interacting elements (i.e., the connectivity between elements in the chemical network) and the parameters that determine those interactions (concentrations, rate constants, binding constants, inhibition/activation parameters, parameters defining environmental conditioning such as temperature or ionic strength dependencies, etc.). We also have an environment **E**, with its set of variables $e_{j}$ and distributions $p(e_{j})$; for instance, the one depicted in Figure 4. We can imagine, now, a variety of systems **S**${}_{i}$ (e.g., a variety of SC), each with its own organization $\Omega_{i}$, which are all “viable” according to some agreed definition of the viability function *V*. These systems **S**${}_{i}$, in other words, have dynamics within the viability region $R_{V}$ (those **S**${}_{i}$ whose dynamics is outside $R_{V}$ are discarded). Each **E-S**${}_{i}$ super system can be subjected to a semantic information analysis, resulting in a variety of semantic information values (in bits), one for each **S**${}_{i}$. Different $\Omega_{i}$ are able to “extract” different amounts of semantic information from the environment **E**. Higher values of semantic information correspond to those systems **S**${}_{i}$, which, in virtue of their mechanisms, connectivity, and parameters (i.e., in virtue of their $\Omega_{i}$), are capable of finer distinctions, perception, or measurements of external variables. It can be said that the corresponding **S**${}_{i}$ have better “knowledge” about the world, or that they better recognize the finer details, the latter being “meaningful” for those systems. Notably, because semantic information is measured based on the system viability, which in turn is determined by the processes within the system and their impact on the property that must be preserved, the above-mentioned operations (perceptions, distinctions, recognition, or measurements) are de facto based on the *perception–action loops* generated by the system itself in a communicative act with the environment. These perception–action loops are evidently well calibrated because they bring about a system’s viability. A posteriori, it can be said that these successful operations have been somehow “selected” by the environmental stimuli among all other potentially occurring dynamics. On the other hand, by analogous reasoning, it can be said that the environment and its stimuli have also been “selected” by certain organizations $\Omega_{i}$, because only for them was there a manner for compensating the perturbations they exerted without disrupting the self-generated viable dynamics. These perception–action loops convey the knowledge that a certain **S**${}_{i}$ has about **E**. Highly performing $\Omega_{i}$ have the network topology and network parameters (which can be thoughts as a set of control knobs) best suited to cope with the external signals (perturbations). Put into these terms, it is still more evident that the concepts of meaning and of being meaningful are relational (Section 2.3) and organizationally based; these features are firmly bound to the coupled **S-E** dynamics.

### 3.4. Can SCs Generate Meaning from the Interaction with Their Environment?

In Section 3.3, we have depicted a scenario where different human-made SCs are placed (situated) in a given environment, and based on a KW semantic information analysis, they could have been ranked on the basis of how much information “makes sense” to them. It can be easily seen that such a knowledge was ultimately *given* to each SC${}_{i}$ by the designer of their $\Omega_{i}$. But can SCs genuinely generate meaning from the interactions engaged with the environment?

A system becomes cognitive, in an autopoietic sense [5,6,108], when it *autonomously selects the stimuli* to which it will react (adapt). As mentioned in Section 3.3, it should display plasticity of its Ω; this means that Ω must change [8]. In the words of Bitbol and Luisi:

“[a] cognitive structure […] selects, and retroactively alters, the *stimuli* to which it is sensitive. By this combination of choice and feed-back, the organic structure determines (in a way *moulds*) its own specific environment; and the environment in turn brings the cognitive organization to its full development. The system and the environment make one another: cognition according to Maturana and Varela is a process of co-emergence” (italics in the original text) [90].

Now, time enters into play, as we argue that the generation of meaning can occur in two different time scales: the evolutionary time and the present (here-and-now) time. Biological systems have self-generated their meanings during evolution. The known biochemical, signaling, and transcriptional networks are the ones that allow cells to make sense of their environment and allow self-maintenance. The meanings they embody are memorized as patterns in Ω. Fabricated SCs that, let us suppose, would self-reproduce without evolution cannot generate new meanings. Their spectrum of meaningful information is and remains the one embodied in their “frozen” Ω—the one imposed in the moment of their fabrication. The difficulty of evolving SCs is particularly evident when they are built by using highly evolved and complex molecules and their corresponding networks of interactions. The latter are not so prone to changes. Vice versa, it would be more plausible to expect forms of self-organization and evolution when other chemicals are employed, such as is the case of allegedly primitive molecules. Clearly, the co-determining role of the environment must be taken into account. In scientific experiments, the environment **E**, as well as the system **S** under inquiry (the SCs), are both designed. While, as mentioned in Section 1, the molecular domain where wetware AL exists allows unbound **S-E** interactions, in a scientific experiment, the environment is also artificial, and this brings about a new problem of complete predictability, which is slightly different in comparison to what has been said before for software AL approaches. The difference, in this case, is that the “rules of the game”, instead of being fixed by the designer, are fixed by physics and chemistry. In conclusion, the generation of meaning in SC evolutionary time is probably a plausible goal, but not easily achievable.

Looking instead at **S-E** interactions in the here-and-now time scale, the requirements of adaptive self-organization (non-destructive changes of Ω to accommodate the perturbation) seem more affordable. Thanks to the peculiarities of all chemical species, and in particular of macromolecules, SC structures leave some room for plasticity when the reactivity landscape explored by the reaction network is not characterized by deep minimums and high energy barriers, but by less pronounced “hilly” profiles. In this permissive scenario, one can imagine a chemical network that, in the short time scale, self-modifies non-destructively upon specific perturbations. For example, a stimulus that causes an increase in reaction rate also increases the concentration of the reaction end product(s). In turn, this will bring about a rate increase in downhill reactions (or rate reduction if the end product inhibits previous reactions). Self-regulating biochemical pathways can be taken as examples of this sort of mechanism. The positive and encouraging message, then, is about learning how to look with fresh eyes at the dynamics of currently achievable SCs. All types of SCs (Figure 2b) can be identified as suitable systems that display plasticity. When macromolecules are used, obvious attention should be paid to 3D structure perturbations (e.g., allosteric regulations). Accordingly, macromolecules might have transistor-like behavior [57] and even simple networks can display multiple dynamical patterns. The changes in Ω, however, must also have a functional consequence on viability—which is a narrower yet necessary constraint.

## 4. Concluding Remarks

SB and Systems Chemistry can provide tools, such as bottom-up SCs, that can be used to investigate semantic information via simulations [103] and experiments (still missing). A first remarkable advantage of these wetware systems when compared to hardware and software is that they are exposed to physical interactions with the environment in which they are situated and that these interactions are, in principle, not programmed by the artificial system designer. Rather, these interactions operate at the level of the structure of the system’s elements and are therefore embodied without the need for symbolic representations [40]. The second equally important advantage of SCs is due to their minimal complexity when compared to actual living wetware systems. The low complexity allows control and programmability when these systems are intended as machine-like agents, and allows a clearer analysis of the system dynamics (without interference from a complex cellular background) when these systems are intended as organism-like agents.

Some directions for future research can be depicted from the presented discussion. Firstly, the investigations based on the KW approach have revealed that modeling SCs as situated agents requires, first of all, that the design of SCs is complemented by the design of their environment. These models explicitly take into account spatial-temporal distributions of environmental variables, noise, and stochastic transition rules. When facing these approaches for the first time, it is easy to identify that probabilistic thinking is a necessary skill. Most of the currently explored SC models (experiments and simulation) do not pay sufficient attention to these aspects. These approaches will help with progressing theories about SC functioning.

Second, it is important to focus on defining, better than we have in this article, the operational criteria to recognize the SC’s traits. Fabricated SCs can be machine-like or organism-like systems, but there is also a territory between these two extremes. Moreover, critical discussions about the utility of applying these categories will help enrich the very concept of SCs and their identity as artificial systems—compared or not to hardware and software ones (Figure 1). Such analyses could be carried out on already-built systems (the ones described in published articles) to verify whether interesting behaviors are already present and take these observations as the starting point for further elaboration.

A third question is about semantic information and the technical definition of meaning, given by MacKay (the selective function that “any event that can be detected by an organism or a machine may exercise […] upon the ensemble of transition probabilities of the behavior of the detector (it operates upon the statistical parameters of the system representing the detector)” [81,109]). In a previous publication, we commented on the MacKay approach [94], but a discussion about how it compares with the semantic information as defined by KW is still missing. The relatively low complexity of SCs, when contrasted with that of biological cells, together with the possibility offered by applying information-theoretical quantities and MC approaches to model their functioning, can favor investigations in this direction. MacKay reasoned in terms of conditional probability matrices, thinking of discrete states. Can simple structures like SCs allow such an analysis?

Finally, we are convinced that understanding the topics presented in this paper can pave the way to a turning point in SC research: expanding the focus from what is now identified with the keyword “minimal life” to what can be labeled with the keyword “minimal cognition”. If this happens, we believe that wetware AL will have a great chance of progressing our understanding of the most important biological phenomena. Moreover, working on the origin of meaning in SCs can favor a discovery about how artificial systems can ground their “symbols” (which are, for SCs, chemical patterns) into something intrinsically meaningful for their existence and dynamics, i.e., into some observer-independent features. It will ultimately represent a new and possibly fruitful approach to facing the symbol-grounding problem [9].

## Figures and Tables

**Figure A1:**
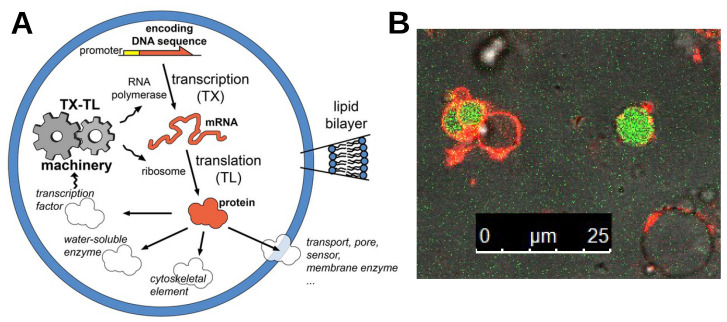
Gene-expressing bottom-up SCs. (**A**) Schematic cartoon of a bottom-up SC based on the encapsulation of biomolecules inside a lipid vesicle. The lipid bilayer provides a semipermeable boundary to the chemical system entrapped in the vesicle lumen. One of the relevant goals in SC research is about synthesizing proteins through gene expression. At this aim, the full TX-TL biochemical machinery (generally obtained from *Escherichia coli*) is entrapped inside lipid vesicles together with DNA sequences encoding for the protein(s) of interest. The produced protein(s) can then act as a transporter, pore, sensor, enzyme, cytoskeletal element, and transcription factor. Reprinted with permission from Ref. [125]. Copyright 2019, John Wiley and Sons. (**B**) Production of green fluorescent protein inside giant vesicles-based SCs. The SC membrane is stained with a red-fluorescent dye; the green fluorescence inside some SCs is due to the in situ synthesized green fluorescent protein. Note that not all SCs are able to produce the fluorescent protein, probably because the preparation method introduces a diversity in the composition of the inner solution because of stochastic effects. The size bar measures 25 μm.

**Figure A2:**
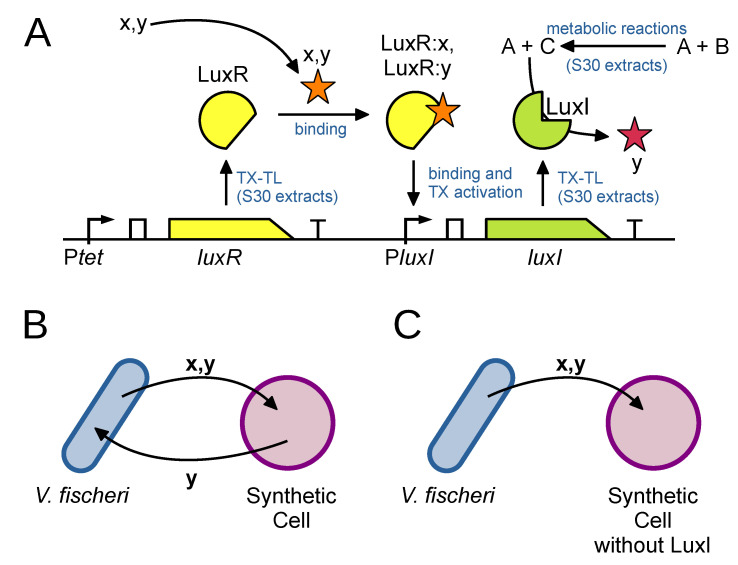
SC–*V. fischeri* bidirectional communication [25]. (**A**) Details about the mechanisms allowing SC to sense molecules x,y (input), and produce y (output) by involving a genetic construct encoding for LuxR (binds x,y and forms a complex that activates transcription downstream the promoter P*luxI*), and LuxI (synthesize y starting from precursors A+C; C is in turn obtained from B thanks to enzymes present in the S30 extract). TX-TL reactions are carried out by RNA polymerase, ribosomes, tRNA, …also present in the S30 extract. Note that x: C8-HLS; y: 3OC6-HSL; A: *S*-adenosylmethionine; B: acetyl-coenzyme A. (**B**) Successful two-way chemical communication between SCs endowed with the genetic construct shown in (**A**) and *V. fischeri*. Bacteria activate biochemiluminescence upon receiving y signal. (**C**) Much less bioluminescence is observed when SCs are endowed with a genetic construct where *luxI* gene is substituted with the one encoding for T7 RNA polymerase. This negative control experiment shows that *V. fischeri* self-activation, via signal y, even if occurring, contributes little to the overall signal measured in (**B**).

## Data Availability

No new data were created or analyzed in this study.

## Abbreviations

The following abbreviations are used in this manuscript:

AI	Artificial Intelligence
AL	Artificial Life
**E**	Environment
*I*	Mutual Information
IoBNT	Internet of Bio-Nano Things
KW	Kolchinsky–Wolpert
MC	Molecular Communication
$R_{V}$	Region of Viability
**S**	System
*S*	Semantic Information (Figure 3C)
SB	Synthetic Biology
SC	Synthetic Cell
**S-E**	Super-system formed by the union of **S** and **E**
TE	Transfer Entropy
TX-TL	Transcription-translation
*V*	Viability; value of the viability function
Ω	Organization

## Appendix A. More Information on Bottom-Up Synthetic Cells

### Appendix A.1. Pioneering Works on Bottom-Up Synthetic Cells

It is now conventional to distinguish approaches to SCs in “top-down” and “bottom-up”. Top-down SCs are those living structures obtained by procedures of modification biological cells. The modification can refer to genetic/metabolic rewiring, addition/deletion of genes, hybridization, combination, artificial control, up to dramatic operations such as the transplantation of a fully synthetic minimal genome [29,30]. These approaches are typical of modern SB, the quite radical bioengineering practice born in the early 2000s [110,111,112]. Bottom-up approaches, on the other hand, have older cultural roots, dating back to the end of the XIX Century, when the first attempts to “synthesize life” from inanimate matter, simulating the spontaneous emergence of life on Earth, were recorded [113]. Stéphen Leduc entitled his 1912 book on similar subjects as *La Biologie Synthétique*. In the 1920s Aleksandr I. Oparin and Jack B. S. Haldane independently concur to formulate hypotheses on abiogenesis. Oparin himself carried out experiments on enzyme-containing cell-like systems, obtained by the coacervation of gum arabic and gelatin, aiming at generating what we would now call (a type of) bottom-up SCs. Much less known is instead the contribution of Thomas Ming Swi Chang, who already in the 1960s explored the possible use of enzyme-filled cellulose nitrate microcapsules in medicine [114,115]. The name given to these structures actually was “artificial cells” and were thought as carriers of enzymes, to compensate malfunctioning metabolic functions. P. L. Luisi, inspired by the theory of autopoiesis [5,116], explored simple chemical systems that could display autopoietic dynamics, such as reverse micelles [117], micelles, and vesicles. Next, enzyme-containing vesicles were employed explicitly aiming at constructing bottom-up synthetic cells (also called semi-synthetic minimal cells) [118,119,120,121]. Additional pioneering work has been reported by David Deamer [122], Jack W. Szostak [123], Tetsuya Yomo [124]. As commented elsewhere [125], the definitive shift toward currently employed strategies and methodologies for bottom-up SCs happened thanks to a series of work published in 2001–2004 [124,126,127,128,129,130].

To date, research on bottom-up SCs is under strong development and many groups work on it, focusing on several different questions, scopes, approaches. Importantly, several types of compartments are employed to build SCs, namely lipid vesicles, fatty acid vesicles, polymer vesicles, coacervates, water-in-oil (w/o) emulsion droplets, water-in-oil-in-water (w/o/w) double emulsion droplets, aqueous two-phase systems and several combinations, including those based on biological and artificial materials (see Figure 2b). The resulting SCs are studied as individuals or grouped together—in tissue-like ensembles. SCs with smaller internal compartments are used as models of eukaryotic cells, or just to achieve specific and more complex behaviors [131]. Interested readers should refer to recent reviews, e.g., [132,133,134,135,136,137,138,139,140,141,142,143,144,145,146,147,148,149].

### Appendix A.2. Synthetic Cells Based on the Encapsulation of Biomacromolecules inside Giant Vesicles: A Popular Model

It seems unquestionable that SCs based on lipid vesicles, i.e., liposomes, are quite relevant cellular models because of their structural similarity with biological cells and membranes. Although it is certainly possible to work with conventional (submicrometer-sized) lipid vesicles, a popular model is based on giant vesicles [150,151], which are cell-sized vesicles. Their usage offers several practical advantages: (i) they are easy to observe by fluorescence microscopy and flow cytometry; (ii) because of their large size, they encapsulate in their lumen a large number of molecules participating to the SC reaction network; (iii) thanks to a relatively novel method—the droplet transfer method [21,129,152,153]—they can be easily formed (within minutes), starting from very small volumes of aqueous solutions, and with rather high entrapment efficiency (e.g., 40% or more); (iv) they can be obtained by microfluidic devices in highly reproducible way.

With respect to the network of reactions taking place inside SCs, and about the functionalization of their lipidic membrane, enzyme-based networks are widely, but not uniquely, used. Purified enzymes (water soluble, membrane-associate, membrane-integral) can be introduced in SCs, following different methods, in order to generate simple or multi-step biochemical pathways. In order to best mimic cells, however, instead of encapsulating the enzymes of interest, SCs are allowed to produce them in situ by TX-TL, i.e., starting from the corresponding genetic sequences. This second approach generates a much more complex chemical network upstream the enzymatic reaction of interest, and requires many additional compounds (RNA polymerase, tRNAs, ribosomes, etc.). However, it also allows introducing regulation and control in SCs, as well as a much more autopoietic-like dynamics. Ideally, the entire set of SC molecules, boundary-ones included, should be generated by such kind of network. Moreover, the TX-TL approach of bottom-up SCs conceptually converges with the top-down “minimal genome” SCs briefly mentioned above [29,30].

A general scheme of gene-expressing bottom-up SCs and their potentialities in terms of SC functions (enzyme catalysis, membrane pores, transporters, sensors, cytoskeletal elements, transcription factors, …) is shown in Figure A1A, while the typical appearance of SCs producing the green fluorescent protein is shown in Figure A1B.

### Appendix A.3. A Specific Example of Communicating SC: Bidirectional Communication between Synthetic Cells and Bacteria [25]

The importance of constructing SCs capable of communicating with each other and with biological cells has been recognized as a relevant goal in the field since 2006, when a theoretical paper about an assay for SCs, similar to the famous Turing test, was presented. The authors discussed it as an operational assay for AL, very much as the original Turing test was for AI [38]. Ben Davis and collaborators firstly reported, in 2009, a simple SC that was able to synthesize in its inner core a sugar-like chemical that, after reaction with borate outside the SC, gave a compound similar to bacterial autoinducer-2. The resulting molecule was anyway perceived as a signal by *Vibrio harveyi* bacteria, which activated a biochemiluminescence response [154]. However, such a simple SC could not show any other complex behavior: it just behaved as a source of signal molecules.

Conversely, gene-expressing SCs have the adequate complexity for achieving more interesting behavior [155]. In particular, we refer to the capacity of sending chemical signals to, and receiving other signals from, biological cells. The group of Sheref Mansy reported in 2017 the first and groundbreaking example of these systems [25]. The work can be considered a proof of principle of SC capabilities in molecular communication, and at the same time—with proper modification—of the “smart” drug delivery scenario depicted in Figure 3A,B. Thanks to a proper design, SCs can perceive signals from their environment and so in-form themselves about a certain situation. The latter is characterized by chemicals which triggers in the SC, thanks to internal mechanism, a specific action devised by the SC designer. It is in this sense that SCs are programmable devices.

The study has been implemented via a *bidirectional* exchange of quorum sensing molecules between bottom-up SCs and *V. fischeri* bacteria (Figure A2). In particular, SCs have been constructed by encapsulating inside lipid vesicles: (a) *E. coli* S30 extracts (competent for carrying out TX-TL processes and other metabolic reactions); (b) a specially made DNA plasmid, and (c) the precursors of acylhomoserine lactones (acyl-HSL, the signaling molecules that SCs will produce). The genetic construct is made of two main elements (Figure A2A): the *luxR* gene that is constitutively expressed by the S30 extract to produce the LuxR receptor, and, downstream to a P*luxR* promoter, the *luxI* gene. The expression of *luxI* occurs only when LuxR binds its cognate signal molecules (indicated as x and y in Figure A2A). Once LuxI (an enzyme) is produced, it catalyzes the synthesis of the signal molecule y from its precursors, already present inside the SC. The overall system, made by SCs and *V. fischeri* cells that communicate, is shown in Figure A2B,C.

*V. fischeri* starts the communication by producing and releasing 3OC6-HSL and C8-HSL, two acyl-HSL species that reach the SCs by diffusion. Once entered in the SCs (because they can cross the lipid membrane), these molecules bind to the LuxR receptor. The LuxR receptor—once bound to HSLs—in turn binds to the P*luxR* promoter, allowing *luxI* expression, and the production of 3OC6-HLS signal molecule from the precursors. 3OC6-HSL molecules cross the SC membrane, are released in the medium, reach *V. fischeri* cells and activate a pathway leading to measurable biochemiluminescence. Importantly, when non-functioning SCs are employed (SCs without the *luxI* gene), much less biochemiluminescence was observed.

As mentioned, the Mansy’s system represents the first proof of concept that SCs can operate according to sophisticated stimulus-response mechanisms while interfaced with biological cells. Their behavior is genetically encoded, because these SCs function thanks to gene expression. If the LuxI element is substituted with another “actuator”, i.e., a protein that performs a certain desired useful operation, SCs can act in controlled manner upon sensing a certain signal in the environment. Such a mechanism paves the way to use SCs as “smart” drug delivery (or drug producing) agents. Studies about how SCs can be employed with respect to their interaction with biological cells, for instance to kill or heal malignant or dangerous cells, are still rare. The concept of “artificial cell” developed by Chang should be recalled again [156], but also highlighting that it refers to systems that, although effective, do not fully share the cell-like organizational complexity typical of SCs, and do not support the sophisticated control mechanisms shown in Figure A1 and Figure A2. Readers interested to SC/biological cell interactions, and their possible use in nanomedicine, can refer to some recent publications [45,47,48,157,158,159,160].
